# Lipoprotein(a) and Adverse Outcomes After Successful Percutaneous Coronary Intervention for Chronic Total Occlusion: A Single-Center Retrospective Cohort Study

**DOI:** 10.3390/jcdd13070320

**Published:** 2026-07-09

**Authors:** Jing Wang, Qiheng Wan, Zehan Huang, Yuqing Huang, Bin Zhang, Song Wen

**Affiliations:** 1 Department of Cardiology, Shenzhen Luohu Hospital Group Luohu People’s Hospital, The Third Affiliated Hospital of Shenzhen University, Shenzhen 518000, China; 2 Department of Cardiology, Guangdong Cardiovascular Institute, Guangdong Provincial People’s Hospital, Guangdong Academy of Medical Sciences, Southern Medical University, Guangzhou 510080, China; 13662056294@163.com (Q.W.);; 3 Department of Cardiology, Fuwai Hospital, National Center for Cardiovascular Diseases, Chinese Academy of Medical Sciences and Peking Union Medical College, Beijing 100037, China

**Keywords:** Lipoprotein(a), percutaneous coronary intervention, chronic total occlusion, outcomes

## Abstract

**Background**: Lipoprotein(a) [Lp(a)] is a genetically determined, atherogenic, and prothrombotic lipoprotein. However, its prognostic value in patients who undergo successful chronic total occlusion (CTO) percutaneous coronary intervention (PCI) remains undefined. **Methods**: This single-center retrospective cohort study included 1509 patients who underwent successful CTO PCI. The primary outcome was cardiovascular death; secondary outcome was major adverse cardiovascular events (MACEs, cardiovascular death or nonfatal myocardial infarction). Multivariable Cox regression and restricted cubic splines (RCS) assessed the association between Lp(a) and outcomes. **Results**: Over median follow-up of 810 days, 53 (3.5%) cardiovascular deaths and 62 (4.1%) MACEs occurred. Each 1-SD increase in log-transformed Lp(a) was associated with a 51% higher risk of cardiovascular death (aHR 1.51, 95% CI 1.11–2.05, *p* = 0.008) and a 44% higher risk of MACEs (aHR 1.44, 95% CI 1.09–1.91, *p* = 0.011). Compared with Lp(a) < 30 mg/dL, Lp(a) ≥ 50 mg/dL conferred a 2.07-fold higher risk of cardiovascular death (95% CI 1.07–4.00, *p* = 0.029) and a 1.94-fold higher risk of MACEs (95% CI 1.07–3.53, *p* = 0.030). RCS analysis demonstrated a linear dose–response relationship between log-transformed Lp(a) and both cardiovascular death (*p* for nonlinearity = 0.653) and MACEs (*p* for nonlinearity = 0.562). The association was modified by age, hypertension, and left ventricular ejection fraction and remained robust in sensitivity analyses. **Conclusions**: In patients undergoing successful CTO PCI, elevated Lp(a) was independently and linearly associated with higher risks of cardiovascular death and MACEs. These findings suggest that Lp(a) may serve as a useful prognostic marker to enhance risk stratification in this high-risk population. Large-scale prospective cohorts are needed to validate these findings before clinical translation can be considered.

## 1. Introduction

Chronic total occlusion (CTO) is present in approximately 15% to 30% of patients undergoing coronary angiography and represents the most technically challenging lesion subset for percutaneous coronary intervention (PCI) [1,2]. Advances in dedicated guidewires, microcatheters, and retrograde approaches have substantially improved procedural success rates for CTO PCI at experienced centers, expanding treatment options for patients with symptomatic coronary artery disease (CAD) [3]. Successful CTO revascularization has been associated with relief of angina, improvement in left ventricular function, and a reduced need for subsequent coronary artery bypass grafting (CABG) [4,5,6,7]. Nevertheless, even after anatomically successful CTO PCI, patients remain at substantial risk for adverse cardiovascular events during long-term follow-up, underscoring the need to identify modifiable and nonmodifiable risk factors that contribute to residual risk in this population [8,9].

Lipoprotein(a) [Lp(a)] is a genetically determined lipoprotein with atherogenic, proinflammatory, and prothrombotic properties. Elevated Lp(a) is a well-established risk factor for atherosclerotic cardiovascular disease (ASCVD), calcific aortic valve stenosis, and incident cardiovascular events in both primary and secondary prevention populations, as demonstrated by large-scale epidemiological and Mendelian randomization studies [10,11,12,13,14,15,16]. However, its prognostic value specifically in patients who have undergone successful CTO PCI remains incompletely defined. Patients undergoing CTO PCI constitute a distinct high-risk cohort characterized by advanced atherosclerotic burden, prolonged ischemic insult, and a prothrombotic milieu, features that could plausibly modify or amplify the risk associated with high Lp(a). Moreover, although contemporary European and American guidelines recommend measuring Lp(a) at least once in every adult, its clinical utility for guiding post-revascularization management in CTO patients is uncertain [17,18].

To address this gap, we conducted a single-center retrospective cohort study of 1509 patients who underwent successful CTO PCI at a high-volume center. We sought to examine the association between Lp(a) concentrations and the long-term risks of cardiovascular death and major adverse cardiovascular events (MACEs). We hypothesized that elevated Lp(a) would be independently associated with increased risks of both outcomes in this high-risk population.

## 2. Methods

### 2.1. Study Design and Population

This single-center retrospective cohort study included 1756 consecutive adult patients who underwent PCI for at least 1 angiographically confirmed CTO lesion at Guangdong Provincial People’s Hospital between February 2011 and December 2023. Patients were excluded if they had procedural failure (*n* = 72), incomplete Lp(a) or essential covariate data (*n* = 79), or were lost to follow-up (*n* = 100). After exclusions, 1509 participants were included in the final analysis.

The study protocol conformed to the principles of the Declaration of Helsinki and was approved by the Institutional Review Board of Guangdong Provincial People’s Hospital. Written informed consent was obtained from all participants. Reporting followed the Strengthening the Reporting of Observational Studies in Epidemiology (STROBE) guidelines to ensure transparency and methodological rigor.

### 2.2. Data Collection and Definitions

CTO PCI was performed in patients with angiographically confirmed CTOs, indicated for refractory symptoms (e.g., angina or dyspnea) despite optimal medical therapy, objective evidence of viable myocardium, or inducible ischemia in the CTO territory. Procedural details have been described previously. Coronary angiography and PCI were performed via femoral, radial, or combined access. Crossing strategies, guide catheters, stent types, and use of intravascular imaging were at the operator’s discretion. All operators were high-volume operators, each having performed >300 CTO PCI procedures in total, with a minimum of 50 CTO PCIs annually as the primary operator. CTO was defined as complete interruption of antegrade blood flow on coronary angiography, with an estimated occlusion duration of ≥3 months [19]. Occlusion duration was determined based on symptom chronology, history of prior MI, and previous angiographic studies. Technical success was defined as achievement of Thrombolysis in Myocardial Infarction (TIMI) grade ≥ 2 antegrade flow in all distal branches ≥ 2.5 mm with residual stenosis < 30% in the target CTO lesion at procedure end. Procedural success was defined as technical success without in-hospital major adverse cardiovascular events (MACEs; i.e., death, MI, or clinically driven target vessel revascularization) [19].

Baseline demographic and clinical characteristics were retrospectively collected for all participants in the study. Demographic information encompassed age, sex, comorbidities, smoking status, and history of prior MI or revascularization (PCI or CABG). Clinical information comprised physical examination findings, laboratory results, imaging data, and discharge medications. Fasting venous blood samples were drawn after ≥12 h of fasting and analyzed at the core laboratory of Guangdong Provincial People’s Hospital under standardized quality control. Measured parameters included complete blood count, total cholesterol (TC), triglycerides (TG), high-density lipoprotein cholesterol (HDL-C), low-density lipoprotein cholesterol (LDL-C), Lp (a), apolipoprotein A1 (ApoA1), ApoB100, fasting blood glucose (FBG), hemoglobin A1c (HbA1c), C-reactive protein (CRP), blood urea nitrogen (BUN), creatinine (Cr), uric acid (UA), alanine aminotransferase (ALT), aspartate aminotransferase (AST), albumin (ALB), creatine kinase (CK), CK-MB, Troponin T (TNT), and NT-pro brain natriuretic peptide (NT-proBNP). Serum Lp(a) concentrations were measured with a latex-enhanced immunoturbidimetric assay on an automated chemistry analyzer (Beckman Coulter AU5800, Brea, CA, USA). Left ventricular ejection fraction (LVEF) was assessed at rest using the modified biplane Simpson’s rule [20]. Diabetes mellitus (DM) was defined by prior diagnosis, use of glucose-lowering agents, or meeting any of the following: FBG ≥ 7.0 mmol/L, HbA1c ≥ 6.5%, or 2 h plasma glucose ≥ 11.1 mmol/L on oral glucose tolerance testing [21]. Hypertension was defined as a prior diagnosis, current antihypertensive treatment, or measured blood pressure ≥ 140/90 mmHg [22].

### 2.3. Follow-Up and Clinical Endpoints

Median follow-up duration was 810 days (interquartile range, 409–1230 days). Follow-up data were obtained through medical record review, outpatient visits, and structured telephone interviews conducted by trained researchers blinded to baseline data. The primary endpoint was cardiovascular mortality. The secondary endpoint was MACEs, defined as cardiovascular death or nonfatal MI. We have clarified that the endpoints were prespecified before data analysis, based on prior CTO PCI prognostic studies and the consensus recommendations. Cardiovascular mortality was defined as death attributable to acute myocardial infarction, sudden cardiac death, heart failure, stroke, or other cardiovascular causes unless a non-cardiovascular cause was unequivocally established through review of hospital records, death certificates, and autopsy reports when available. Nonfatal MI was defined according to contemporary guidelines as a rise in cardiac troponin accompanied by typical chest pain, serial electrocardiographic changes, identification of intracoronary thrombus on angiography or autopsy, or imaging evidence of new loss of viable myocardium or new regional wall motion abnormality [20]. All endpoint events were adjudicated by two independent physicians who were blinded to baseline Lp(a) values.

### 2.4. Statistical Analysis

Normality of continuous variables was assessed with the Shapiro–Wilk test. Continuous variables were presented as median (interquartile range) and categorical variables as frequency (percentage). Between-group comparisons were performed using the Wilcoxon rank-sum test for continuous variables and the chi-square test or Fisher’s exact test for categorical variables.

Kaplan–Meier survival curves with log-rank tests illustrated the incidence of cardiovascular death and MACEs across Lp(a) groups. Multivariable Cox proportional hazards models estimated hazard ratios (HRs) and 95% confidence intervals (CIs) for the associations between log-transformed Lp(a) and clinical outcomes, due to its skewed distribution. The population was then stratified by clinically relevant Lp(a) thresholds of 30 mg/dL and 50 mg/dL, thresholds associated with increased cardiovascular risk. Three models were constructed: Model 1 was unadjusted; Model 2 adjusted for age and sex; Model 3 additionally adjusted for smoking, hypertension, diabetes, dyslipidemia, prior MI, prior stroke, target CTO artery, multi-vessel disease, TG, HDL-C, FBG, HbA1c, creatinine, CRP, LVEF, statin use, and dual antiplatelet therapy. Covariates included in the multivariable Cox proportional hazards models were prespecified on the basis of established clinical relevance and previous prognostic studies in CTO PCI populations. The proportional hazards assumption was verified using Schoenfeld residuals, and no significant violations were detected. Potential dose–response associations between log-transformed Lp(a) and outcomes were examined using restricted cubic splines with three knots placed at the 30th, 60th, and 90th percentiles.

We conducted exploratory analyses, including testing for interaction, to assess the association between log-transformed Lp(a) and outcomes across subgroups defined by sex, age (<60 vs. ≥60 years), smoking, hypertension, diabetes, prior MI, LDL-C (<2.6 vs. ≥2.6 mmol/L), and LVEF (<50% vs. ≥50%). Sensitivity analyses assessed the robustness of primary findings by (1) excluding patients with prior stroke; (2) excluding patients with follow-up <60 days; (3) excluding patients with prior CABG; and (4) re-fitting Cox Model 3 with additional adjustment for baseline ApoB100 and ApoA1.

A two-sided *p* < 0.05 was considered statistically significant. All analyses were performed using R software, version 4.1.2 (R Foundation for Statistical Computing).

## 3. Results

### 3.1. Baseline Characteristics

The study cohort comprised 1509 participants, with a median age of 59.0 (IQR, 52.0–67.0) years; 1382 (91.6%) were male. Baseline characteristics stratified by Lp(a) tertiles were summarized in Table 1. Compared with patients in the lowest Lp(a) tertile (T1), those in higher tertiles had a higher prevalence of multi-vessel disease (86.9% vs. 89.8% vs. 91.8%, *p* = 0.037) but a lower prevalence of DM (46.5% vs. 38.1% vs. 34.8%, *p* < 0.001). Laboratory profiling revealed that higher Lp(a) tertiles were characterized by elevated levels of CRP, LDL-C, TC, HDL-C, platelet count, NT-proBNP, troponin T, and ApoB100, alongside lower TG, ApoA1, hemoglobin, FBG, HbA1c, and ALB (all *p* < 0.05). No significant differences were observed across tertiles in the use of statins, β-blockers, dual antiplatelet therapy, calcium channel blockers, or angiotensin-converting enzyme inhibitor (ACEI)/angiotensin II receptor blocker (ARB) (all *p* > 0.05).

### 3.2. Association of Lp(a) with Clinical Outcomes in Patients with Successful CTO PCI

**Figure 1:**
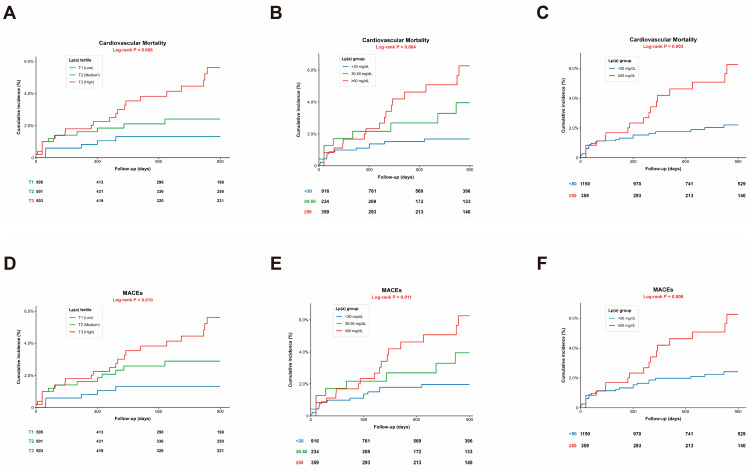
Kaplan–Meier curves of the cumulative incidence of cardiovascular mortality and MACEs according to Lp(a). Abbreviations: Lp(a), Lipoprotein(a); MACEs, major adverse cardiac events. (**A**) Cumulative incidence of cardiovascular mortality according to Lp(a) tertiles (T1, low; T2, medium; T3, high); (**B**) Cumulative incidence of cardiovascular mortality according to Lp(a) groups (<30, 30–50, and ≥50 mg/dL); (**C**) Cumulative incidence of cardiovascular mortality comparing Lp(a) <50 mg/dL vs. ≥50 mg/dL; (**D**) Cumulative incidence of MACEs according to Lp(a) tertiles (T1, low; T2, medium; T3, high); (**E**) Cumulative incidence of MACEs according to Lp(a) groups (<30, 30–50, and ≥50 mg/dL); and (**F**) Cumulative incidence of MACEs comparing Lp(a) <50 mg/dL vs. ≥50 mg/dL.

**Figure 2:**
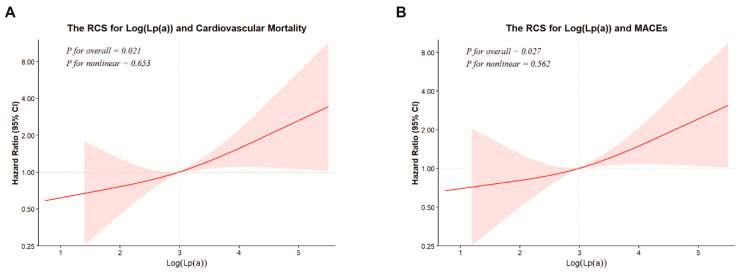
(**A**). RCS analysis of the log-transformed Lp(a) for incidence of cardiovascular mortality; (**B**). RCS analysis of the log-transformed Lp(a) for incidence of MACEs. Abbreviations: Lp(a), Lipoprotein(a); MACEs, major adverse cardiac events.

During a median follow-up of 810 (IQR: 409–1230) days, 53 (3.5%) cardiovascular deaths and 62 (4.1%) MACEs were recorded. Figure 1 shows Kaplan–Meier curves for the cumulative incidence of cardiovascular death and MACE according to Lp(a) tertiles. After adjusting for potential confounding variables, elevated Lp(a) levels were independently and significantly associated with an increased risk of cardiovascular death (Table 2). Each 1-standard deviation (SD) increase in log-transformed Lp(a) was associated with a 51% higher risk of cardiovascular mortality [adjusted hazard ratio (aHR) 1.51, 95% CI 1.11–2.05, *p* = 0.008]. When Lp(a) was analyzed by tertiles, a graded association was observed. Compared with the lowest tertile (T1), patients in the highest tertile (T3) had a 2.43-fold higher risk of cardiovascular death (aHR 2.43, 95% CI 1.14–5.19, *p* = 0.022). Using guideline-informed thresholds, patients with Lp(a) ≥ 50 mg/dL had a 2.07-fold increased cardiovascular mortality risk relative to those with Lp(a) < 30 mg/dL (aHR 2.07, 95% CI 1.07–4.00, *p* = 0.029) and a 1.91-fold increased risk relative to those with Lp(a) < 50 mg/dL (aHR 1.91, 95% CI 1.04–3.50, *p* = 0.038). RCS analysis revealed a linear association between log-transformed Lp(a) and cardiovascular mortality (*p* for nonlinearity = 0.653) (Figure 2).

Results for MACEs mirrored those for cardiovascular mortality (Table 2). In the fully adjusted model (Model 3), each 1-SD increase in log-transformed Lp(a) was associated with a 44% higher risk of MACEs (aHR 1.44, 95% CI 1.09–1.91, *p* = 0.011). Compared with T1, patients in T3 had a 2.40-fold higher risk of MACEs (aHR 2.40, 95% CI 1.16–4.95, *p* = 0.018). Additionally, patients with Lp(a) ≥ 50 mg/dL had a 1.94-fold higher risk of MACEs compared with those with Lp(a) < 30 mg/dL (aHR 1.94, 95% CI 1.07–3.53, *p* = 0.030) and a 1.88-fold higher risk compared with those with Lp(a) < 50 mg/dL (aHR 1.88, 95% CI 95% CI 1.08–3.28, *p* = 0.027). RCS analysis revealed a linear association between log-transformed Lp(a) and MACEs (*p* for nonlinearity = 0.562, Figure 2).

### 3.3. Subgroup Analyses

Stratified analyses were performed to evaluate the association between Lp(a) and cardiovascular mortality across various subgroups (Table 3). The association between Lp(a) and cardiovascular death was more pronounced in males, patients aged <60 years, and those with hypertension, diabetes, no prior MI, LVEF ≥ 50%, LDL-C < 2.6 mmol/L, or no history of smoking (Table 3). The association between log-transformed Lp(a) and cardiovascular death was significantly modified by age (*p* for interaction < 0.001), hypertension status (*p* for interaction = 0.033), and LVEF (*p* for interaction = 0.005). Similarly, stratified analyses were conducted to assess the association of Lp(a) with MACEs across subgroups (Table S1).

### 3.4. Sensitivity Analyses

Multiple sensitivity analyses confirmed the robustness of the primary findings (Tables S2–S5). After sequentially excluding patients with follow-up <60 days (*n* = 1476), those with prior CABG (*n* = 1474), and those with prior stroke (*n* = 1436), and after additional adjustment for ApoB100 and ApoA1, the positive associations between Lp(a) and both cardiovascular death and MACEs remained statistically significant and materially unchanged. Across all sensitivity models, each 1-SD increase in log-transformed Lp(a) remained significantly associated with cardiovascular death (aHRs 1.55–1.69, all *p* ≤ 0.008) and with MACEs (aHRs 1.47–1.56, all *p* ≤ 0.011), supporting the stability and reliability of the primary findings.

## 4. Discussion

In this single-center retrospective cohort study of 1509 patients who underwent successful CTO PCI, we found that elevated Lp(a) concentrations were independently and linearly associated with an increased risk of cardiovascular death and MACEs over a median follow-up of 810 days. After comprehensive adjustment for confounding factors, each 1-SD increase in log-transformed Lp(a) was associated with a 51% higher risk of cardiovascular death and a 44% higher risk of MACEs. And patients with Lp(a) ≥ 50 mg/dL had a 1.91-fold increased cardiovascular mortality risk and a 1.88-fold increased MACEs risk relative to those with Lp(a) < 50 mg/dL. Notably, the prognostic value of Lp(a) was modified by age, hypertension, and LVEF, with stronger risk increments observed among younger patients (<60 years), those with hypertension, and individuals with preserved LVEF (≥50%). To our knowledge, this is among the first studies to specifically characterize the prognostic value of Lp(a) in a well-defined cohort of patients after successful CTO revascularization. Although elevated Lp(a) was independently associated with adverse outcomes, this observational study was not designed to evaluate whether Lp(a) improves risk prediction beyond established clinical variables. Whether Lp(a) provides meaningful incremental discrimination or reclassification in patients undergoing CTO PCI requires dedicated prognostic modeling in future studies.

Our findings align with a large body of evidence from population-based cohorts, Mendelian randomization studies, and secondary prevention trials, all demonstrating that Lp(a) is an independent, causal risk factor for ACSVD and cardiovascular death [10,11,12,13,14,15,16]. A Japanese study of 1336 patients with CAD who received statin therapy after PCI showed a significant association between log-transformed Lp(a) and increased MACEs (aHR 1.28, 95%CI 1.04–1.58, *p* = 0.018) [23]. In a study of 4469 patients with prior cardiovascular events who underwent PCI, followed for a mean of 5.0 years, Lp(a) ≥ 30 mg/dL remained an independent risk factor for MACCEs (aHR 1.24, 95%CI 1.07–1.44, *p* = 0.006), all-cause death (aHR 1.45, 95%CI 1.02–2.04, *p* = 0.037), and cardiac death (aHR 1.72, 95%CI 1.11–2.68, *p* = 0.016) after multivariable adjustment [24]. A meta-analysis of 11 cohorts comprising 27,618 patients who underwent PCI with drug-eluting stents reported that high Lp(a) levels were associated with increased risks of MACEs [odds ratio (OR) 1.25, 95% CI 1.09–1.42], MI (OR 1.75, 95% CI 1.08–2.83), stroke (OR 1.28, 95% CI 1.04–1.59), cardiovascular death (OR 1.37, 95% CI 1.02–1.83), and all-cause mortality (OR 1.29, 95% CI 1.04–1.59) [25]. A South Korean study of 12,064 patients who underwent PCI during a median follow-up of 7.4 years found that Lp(a) ≥ 30 mg/dL was significantly associated with recurrent ischemic events (aHR 1.17, 95%CI 1.05–1.30, *p* = 0.005) and repeat revascularization (aHR 1.13, 95% CI 1.02–1.25, *p* = 0.022) [16]. A multicenter prospective study of 4078 patients with stable CAD undergoing PCI during an average of 4.9 years of follow-up demonstrated that patients with Lp(a) > 30 mg/dL had a 2.1-fold increased risk of cardiovascular events compared with those with Lp(a) < 15 mg/dL, and each 1-SD increment in Lp(a) was associated with a 30% increase in cardiovascular events risk [26]. Our study extends these observations to the specific context of CTO PCI. Patients with CTO represent a distinct phenotype characterized by prolonged myocardial ischemia, extensive coronary atherosclerotic burden, and a prothrombotic milieu resulting from stagnant flow in the occluded vessel and collateral circulation. Despite successful recanalization, these patients face substantial residual risk. In our cohort, the event rates for cardiovascular death and MACEs were 3.5% and 4.1%, respectively, over a median follow-up of approximately 2.2 years, underscoring the need for refined risk stratification. We demonstrated that each 1-SD increment in log-transformed Lp(a) conferred a 51% higher risk of cardiovascular death and a 44% higher risk of MACEs. When applying guideline-recommended thresholds (≥50 mg/dL), patients had a 2.07-fold higher risk of cardiovascular death and a 1.94-fold higher risk of MACEs compared with those with Lp(a) < 30 mg/dL. These effect sizes are comparable to or larger than those reported in contemporary post-PCI populations. This larger effect size may reflect the unique pathophysiology of CTO, including prolonged ischemic insult, extensive atherosclerotic burden, and prothrombotic milieu, which may amplify Lp(a)-mediated risk.

Our subgroup analyses revealed several noteworthy findings. The association between Lp(a) and cardiovascular death was significantly modified by age, with a more pronounced effect in patients younger than 60 years. This finding aligns with the concept that genetic determinants of Lp(a) exert their greatest relative risk in younger individuals, before competing risks from traditional risk factors accumulate. In older patients, the background risk from advancing age, longer exposure to hypertension, and cumulative burden of diabetes may attenuate the incremental prognostic impact of a single genetic factor [27]. Nevertheless, Lp(a) remained a significant predictor even in the elderly, emphasizing its importance across the age spectrum. We also observed significant effect modification by hypertension and LVEF. The markedly elevated hazard ratio observed in patients with LVEF ≥ 50% compared with those with LVEF < 50% may seem counterintuitive; however, this finding may be explained by competing risks. In patients with reduced LVEF, competing risks from heart failure and arrhythmic death may overshadow Lp(a)-mediated atherothrombotic risk; however, this interpretation is post hoc and requires confirmation [20]. Conversely, in patients with LVEF ≥ 50%, whose risk of cardiovascular death is more directly related to recurrent atherothrombotic events, Lp(a) emerges as a more dominant risk factor [28]. Furthermore, Lp(a) is not known to directly impair myocardial contractility; its effects are mediated through vascular and valvular pathways. Alternatively, patients with reduced LVEF may have a greater burden of irreversible myocardial damage, rendering them less susceptible to Lp(a)-mediated ischemic events. Thus, in the setting of already compromised LVEF, Lp(a) may be a less potent predictor than clinical markers of heart failure severity. The observed interaction with hypertension raises the possibility of biological synergy: hypertension induces endothelial dysfunction and increases vascular wall shear stress, conditions that may potentiate the proatherogenic, prothrombotic, and proinflammatory effects of Lp(a) [29]. In addition, oxidized phospholipids carried by Lp(a) are known to promote vascular inflammation and calcification, processes that may be accelerated in the hypertensive milieu. Nevertheless, this mechanistic interpretation remains speculative and cannot be directly evaluated with the present observational data. These subgroup analyses were exploratory and should not be interpreted as definitive. The modest event counts within several subgroups limit statistical power, and the observed interactions may reflect chance rather than true biological effect modification. Independent validation in larger cohorts is required before these findings can inform clinical decision-making.

Several mechanisms may underlie the observed association between Lp(a) and adverse outcomes after CTO PCI. First, Lp(a) promotes endothelial dysfunction, oxidative stress, and inflammatory cell recruitment within the arterial wall, thereby accelerating atherosclerotic plaque progression and destabilization [30,31]. Second, the prothrombotic properties of Lp(a), mediated through its homology with plasminogen and inhibition of fibrinolysis, may predispose to stent thrombosis and recurrent coronary events in the peri-procedural and long-term phases [32]. Third, Lp(a) has been implicated in microvascular dysfunction and impaired collateral circulation, factors that are particularly relevant in CTO pathophysiology [33]. Fourth, Lp(a) has been implicated in calcific aortic valve stenosis and myocardial fibrosis, which may contribute to long-term cardiovascular mortality independent of recurrent coronary events [34]. The linear dose–response relationship observed in our RCS analyses, with no apparent threshold effect, supports the concept that Lp(a)-mediated risk operates across the entire concentration spectrum, consistent with recent genetic evidence favoring a continuous causal relationship.

Our findings have substantial clinical implications. Current guidelines recommend universal Lp(a) screening at least once in adulthood to refine cardiovascular risk stratification. Our data support integrating Lp(a) assessment into the routine evaluation of patients undergoing CTO PCI, as it provides prognostic information beyond traditional risk factors and may identify a subgroup of patients who would benefit from more intensive surveillance or targeted therapies. Although potent Lp(a)-lowering agents such as pelacarsen and olpasiran have shown promising efficacy in reducing Lp(a) concentrations by 80% to 95%, their effect on hard cardiovascular endpoints is currently being evaluated in phase 3 trials. Our findings suggest that patients with elevated Lp(a) after successful CTO PCI may represent a particularly compelling target population for such interventions, given their high residual risk and the anatomical complexity of their coronary disease.

Several limitations of this study merit consideration. First, the single-center design and the predominantly male cohort (91.6%), reflecting the well-documented sex distribution in CTO PCI registries, limit the generalizability of our findings to women, other ethnic groups, and health care settings with different patient demographics or procedural volumes. External validation in multi-center, multi-ethnic cohorts is therefore warranted. Second, although we adjusted for a wide range of clinically relevant covariates, residual confounding from unmeasured or imprecisely measured factors, including dietary patterns, physical activity, genetic predisposition, and longitudinal lipid management, cannot be excluded. Notably, we lacked detailed data on statin intensity, ezetimibe or PCSK9 inhibitor use, and on-treatment LDL-C levels during follow-up. Given the correlation between Lp(a) and LDL-C, and the possibility that more intensive lipid-lowering therapy may have been preferentially prescribed to patients with higher LDL-C, unmeasured confounding by lipid treatment intensity remains a concern. Third, Lp(a) was measured only at baseline using a mass-based immunoturbidimetric assay, which is susceptible to isoform-dependent variability. While Lp(a) levels are largely genetically determined and stable in adulthood, we were unable to assess the impact of temporal changes in Lp(a) or to account for apolipoprotein(a) isoform size. Molar units (nmol/L), currently recommended for standardized reporting, would facilitate cross-cohort comparability in future investigations. Fourth, the modest number of events limited statistical precision, particularly for subgroup analyses. With 17 covariates in the fully adjusted model, the events-per-variable ratio of approximately 3.1 for cardiovascular death falls below the conventional threshold of 10. Although the consistency of effect estimates across sequential models and sensitivity analyses supports the robustness of the primary findings, cautious interpretation is warranted, and these results should be regarded as hypothesis-generating. Fifth, our analysis was restricted to patients with successful revascularization, excluding those with procedural failure, which may have introduced selection bias and limits extrapolation to patients in whom CTO PCI was unsuccessful or not attempted. Sixth, the study spanned a 12-year period, during which CTO PCI techniques, procedural success, operator experience, and secondary prevention strategies have evolved substantially. These temporal changes may have influenced both procedural outcomes and long-term event rates. Although we adjusted for medication use at discharge, we could not fully account for changes in practice patterns over time. Future studies should examine whether the prognostic value of Lp(a) remains consistent across different eras of CTO PCI. Finally, the median follow-up of 810 days, while adequate for detecting early cardiovascular events, may underestimate the long-term prognostic implications of Lp(a) in this population. Despite these limitations, the consistency of our findings across multiple sensitivity analyses and their alignment with the established causal role of Lp(a) in atherosclerotic cardiovascular disease from Mendelian randomization studies lend support to the observed associations.

## 5. Conclusions

Among patients undergoing successful CTO PCI, elevated Lp(a) was independently associated with increased risks of cardiovascular death and MACE, with a linear dose–response relationship that remained robust across extensive multivariable and sensitivity analyses. These findings suggest that Lp(a) measurement may serve as a useful prognostic marker to enhance risk stratification in this patient population and may aid in identifying high-risk candidates for emerging Lp(a)-targeted therapies. Prospective studies and randomized controlled trials will be required to establish whether Lp(a) reduction confers improved clinical outcomes in this population.

## Figures and Tables

**Table 1:** Baseline characteristics grouped by Lp(a) levels.

Variables	All (*n* = 1509)	T1 (<10.50 mg/dL, *n* = 505)	T2 (10.50–33.27 mg/dL, *n* = 501)	T3 (>33.27 mg/dL, *n* = 503)	*p* Value
Age, years	59.0 (52.0, 67.0)	59.0 (51.0, 66.0)	60.0 (53.0, 67.0)	59.0 (52.0, 67.0)	0.4
Male, *n* (%)	1382 (91.6%)	471 (93.3%)	448 (89.4%)	463 (92.0%)	0.081
SBP, mmHg	130.0 (119.0, 143.0)	130.0 (118.0, 142.0)	130.0 (119.0, 143.0)	130.0 (119.0, 143.0)	>0.9
DBP, mmHg	78.0 (70.0, 85.0)	78.0 (70.00, 86.0)	78.0 (70.0, 86.0)	77.0 (69.0, 84.0)	0.2
Heart rate, beats/min	74.0 (67.0, 82.0)	74.0 (68.0, 82.0)	74.0 (66.0, 81.0)	74.0 (67.0, 82.0)	0.5
Current smoker, *n* (%)	588 (39.0%)	203 (40.2%)	185 (36.9%)	200 (39.8%)	0.5
Hypertension, *n* (%)	899 (59.6%)	314 (62.2%)	289 (57.7%)	296 (58.8%)	0.3
Diabetes, *n* (%)	601 (39.8%)	235 (46.5%)	191 (38.1%)	175 (34.8%)	<0.001
Dyslipidemia, *n* (%)	423 (28.0%)	132 (26.1%)	134 (26.7%)	157 (31.2%)	0.15
Prior MI, *n* (%)	234 (15.5%)	71 (14.1%)	84 (16.8%)	79 (15.7%)	0.5
Prior PCI, *n* (%)	753 (49.9%)	254 (50.3%)	275 (54.9%)	224 (44.5%)	0.004
Prior CABG, *n* (%)	35 (2.3%)	8 (1.6%)	10 (2.0%)	17 (3.4%)	0.14
Prior stroke, *n* (%)	73 (4.8%)	27 (5.3%)	23 (4.6%)	23 (4.6%)	0.8
**Laboratory results**					
Hb, g/L	135.0 (125.0, 146.0)	137.0 (128.0, 148.0)	134.0 (122.0, 146.0)	134.0 (123.0, 144.0)	<0.001
WBC, 10^9^/L	6.96 (5.84, 8.25)	6.84 (5.85, 8.21)	7.03 (5.87, 8.26)	6.99 (5.77, 8.31)	0.7
PLT, 10^9^/L	217.0 (180.0, 263.0)	209.0 (176.0, 261.0)	215.0 (182.0, 256.0)	224.0 (182.0, 272.0)	0.013
LDL, mmol/L	2.39 (1.87, 2.95)	2.14 (1.72, 2.74)	2.47 (1.99, 3.05)	2.53 (2.05, 3.10)	<0.001
HDL, mmol/L	0.93 (0.82, 1.07)	0.91 (0.81, 1.04)	0.94 (0.81, 1.06)	0.94 (0.83, 1.09)	0.034
TC, mmol/L	3.87 (3.20, 4.60)	3.65 (3.01, 4.37)	3.94 (3.30, 4.75)	4.09 (3.42, 4.89)	<0.001
TG, mmol/L	1.47 (1.06, 2.09)	1.53 (1.13, 2.64)	1.50 (1.03, 1.99)	1.41 (1.04, 1.89)	<0.001
Lp(a), mg/dL	19.70 (7.50, 46.00)	5.40 (5.00, 7.50)	19.80 (14.30, 26.50)	67.60 (46.00, 98.90)	<0.001
ApoA1, g/L	1.11 (1.01, 1.24)	1.14 (1.03, 1.25)	1.10 (1.01, 1.20)	1.12 (0.99, 1.26)	0.030
ApoB100, g/L	0.75 (0.59, 0.91)	0.67 (0.54, 0.84)	0.79 (0.63, 0.91)	0.79 (0.65, 0.96)	<0.001
FBG, mmol/L	5.50 (4.85, 6.90)	5.68 (4.96, 7.12)	5.41 (4.81, 6.92)	5.44 (4.83, 6.59)	0.009
HbA1c, %	6.10 (5.70, 6.80)	6.19 (5.70, 7.00)	6.04 (5.60, 6.70)	6.10 (5.70, 6.60)	0.047
NT-proBNP, pg/mL	229.40 (73.40, 749.70)	168.60 (67.50, 544.80)	251.30 (87.20, 896.70)	271.90 (73.30, 873.40)	<0.001
TNT, pg/mL	17.20 (9.70, 41.35)	15.80 (9.60, 29.50)	18.00 (10.00, 45.40)	19.40 (9.80, 47.90)	0.006
CK, U/L	87.00 (63.00, 122.00)	89.00 (64.00, 116.00)	84.00 (62.00, 119.00)	87.00 (63.00, 128.00)	0.2
CK-MB, U/L	10.20 (10.00, 13.00)	10.40 (10.00, 13.20)	10.10 (10.00, 13.00)	10.30 (10.00, 13.00)	0.4
ALT, U/L	22.00 (16.00, 32.00)	22.94 (16.00, 31.00)	23.00 (16.00, 32.00)	22.00 (15.00, 32.00)	0.6
AST, U/L	22.00 (18.00, 27.00)	22.00 (18.00, 25.00)	22.00 (18.00, 27.00)	22.00 (18.00, 28.00)	0.6
CRP, mg/L	1.98 (0.50, 6.20)	1.20 (0.50, 4.76)	2.45 (0.50, 6.30)	2.38 (0.50, 8.50)	<0.001
UA, μmol/L	404.60 (337.00, 476.40)	403.10 (345.00, 467.00)	404.60 (333.50, 476.40)	404.90 (340.60, 484.60)	0.8
Creatinine, µmol/L	84.00 (72.85, 100.10)	83.30 (72.85, 97.50)	83.11 (72.28, 99.40)	85.21 (73.09, 104.66)	0.15
BUN, µmol/L	5.59 (4.51, 6.83)	5.59 (4.55, 6.84)	5.59 (4.47, 6.62)	5.60 (4.60, 7.29)	0.2
ALB, g/L	38.80 (36.56, 41.00)	39.41 (37.40, 41.74)	38.79 (36.20, 40.90)	38.10 (35.80, 40.50)	<0.001
LVEF, %	58.00 (46.00, 63.00)	59.00 (46.00, 64.00)	57.00 (48.00, 62.00)	58.00 (45.00, 64.00)	0.13
**Angiographic characteristics**					
Target CTO artery					0.7
RCA, *n* (%)	857 (56.8%)	280 (55.4%)	283 (56.5%)	294 (58.4%)	
LAD, *n* (%)	711 (47.1%)	243 (48.1%)	234 (46.7%)	233 (46.3%)	
LCX, *n* (%)	197 (13.1%)	68 (13.5%)	59 (11.8%)	70 (13.9%)	
LM, *n* (%)	3 (0.2%)	0 (0.0%)	2 (0.4%)	1 (0.2%)	
Multi-vessel disease, *n* (%)	1351 (89.5%)	439 (86.9%)	450 (89.8%)	462 (91.8%)	0.037
**Medications**					
DAPT, *n* (%)	1473 (97.6%)	493 (97.6%)	488 (97.4%)	492 (97.8%)	>0.9
Statin, *n* (%)	1490 (98.7%)	500 (99.0%)	491 (98.0%)	499 (99.2%)	0.2
β-Blocker, *n* (%)	1208 (80.1%)	403 (79.8%)	392 (78.2%)	413 (82.1%)	0.3
CCB, *n* (%)	317 (21.0%)	104 (20.6%)	108 (21.6%)	105 (20.9%)	>0.9
ACEI/ARB, *n* (%)	898 (59.5%)	301 (59.6%)	295 (58.9%)	302 (60.2%)	>0.9

Abbreviations: LVEF, left ventricular ejection fraction; PCI, percutaneous coronary intervention; CABG, coronary artery bypass grafting; SBP, systolic blood pressure; DBP, diastolic blood pressure; MI, myocardial infarction; TC, total cholesterol; HDL, high-density lipoprotein cholesterol; LDL, low-density lipoprotein cholesterol; TG, triglycerides; ApoA1, apolipoprotein A1; ApoB100, apolipoprotein B100; FBG, fasting blood glucose; CRP, C-reactive protein; BUN, blood urea nitrogen; UA, uric acid; HbA1c, hemoglobin A1c; Hb, hemoglobin; WBC, white blood cell; PLT, platelet; ALT, Alanine Aminotransferase; BNP, brain natriuretic peptide; TNT, Troponin T; Lp(a), Lipoprotein(a); CK, creatine kinase; CK-MB, creatine kinase-MB; AST, aspartate aminotransferase; ALB, albumin; RCA, right coronary artery; LAD, left Anterior descending artery; LCX, left circumflex artery; LM, left main coronary artery; DAPT, dual antiplatelet therapy; CCB, calcium channel blocker; ACEI, angiotensin-converting enzyme inhibitor; ARB, angiotensin II receptor blocker.

**Table 2:** Associations between Lp(a) and clinical outcomes in participants with successful CTO PCI.

	Model 1		Model 2		Model 3	
	HR (95%CI)	*p*-Value	HR (95%CI)	*p*-Value	HR (95%CI)	*p*-Value
**Cardiovascular mortality**						
Per-SD increase in Log(Lp(a))	1.51 (1.14–2.01)	0.004	1.53 (1.15–2.04)	0.004	1.51 (1.11–2.05)	0.008
T1	Reference		Reference		Reference	
T2	1.47 (0.66–3.23)	0.344	1.44 (0.65–3.17)	0.371	1.31 (0.58–2.96)	0.512
T3	2.54 (1.23–5.26)	0.012	2.58 (1.25–5.33)	0.011	2.43 (1.14–5.19)	0.022
Lp(a)						
<30 mg/dL	Reference		Reference	0.512	Reference	
30–50 mg/dL	1.77 (0.88–3.56)	0.111	1.70 (0.85–3.43)	0.135	1.32 (0.63–2.75)	0.466
≥50 mg/dL	1.99 (1.07–3.69)	0.029	2.09 (1.13–3.89)	0.019	2.07 (1.07–4.00)	0.029
Lp(a)						
<50 mg/dL	Reference		Reference	0.512	Reference	
≥50 mg/dL	1.69 (0.96–2.99)	0.069	1.80 (1.02–3.18)	0.043	1.91 (1.04–3.50)	0.038
**MACEs**						
Per-SD increase in Log(Lp(a))	1.47 (1.13–1.91)	0.005	1.49 (1.14–1.94)	0.004	1.44 (1.09–1.91)	0.011
T1	Reference		Reference		Reference	
T2	1.64 (0.78–3.42)	0.189	1.62 (0.78–3.39)	0.199	1.57 (0.74–3.34)	0.240
T3	2.56 (1.28–5.11)	0.008	2.61 (1.31–5.21)	0.007	2.40 (1.16–4.95)	0.018
Lp(a)						
<30 mg/dL	Reference		Reference	0.512	Reference	
30–50 mg/dL	1.43 (0.73–2.82)	0.300	1.39 (0.70–2.73)	0.346	1.12 (0.55–2.28)	0.762
≥50 mg/dL	1.93 (1.10–3.40)	0.023	2.05 (1.16–3.61)	0.013	1.94 (1.07–3.53)	0.030
Lp(a)						
<50 mg/dL	Reference		Reference	0.512	Reference	
≥50 mg/dL	1.76 (1.04–2.98)	0.037	1.88 (1.10–3.19)	0.020	1.88 (1.08–3.28)	0.027

Model 1 adjust for: None. Model 2 adjust for: age and sex. Model 3 adjust for: age, sex, smoking, hypertension, diabetes, dyslipidemia, prior MI, prior stroke, target CTO artery, multi-vessel disease, statin, dual antiplatelet therapy, LVEF, creatinine, TG, HDL-C, CRP, FBG, and HbA1c. MACEs were defined as a composite of cardiovascular mortality and nonfatal myocardial infarctions. HR, hazard ratio; CI, confidence interval; Lp(a), Lipoprotein(a); CTO, chronic total occlusion; PCI, percutaneous coronary intervention.

**Table 3:** Subgroup analysis of association of Lp(a) and cardiovascular mortality in participants with successful CTO PCI.

Subgroups		Cardiovascular Mortality	*n*	HR (95% CI)	*p* Value	*p* for Interaction
Sex	Male	46	1382	1.69 (1.20–2.36)	0.002	0.234
	Female	7	127	0.56 (0.23–1.37)	0.203	
Age	<60 years	17	765	2.02 (1.15–3.57)	0.015	<0.001
	≥60 years	36	744	1.50 (1.00–2.25)	0.048	
Current Smoking	Yes	21	588	1.60 (0.95–2.69)	0.076	0.902
	No	32	921	1.54 (1.01–2.36)	0.046	
Prior MI	Yes	16	234	1.84 (0.78–4.34)	0.161	0.990
	No	37	1275	1.56 (1.09–2.22)	0.015	
Diabetes	Yes	30	601	1.72 (1.10–2.70)	0.017	0.636
	No	23	908	1.48 (0.93–2.37)	0.098	
Hypertension	Yes	40	899	2.01 (1.37–2.94)	<0.001	0.033
	No	13	610	0.84 (0.40–1.78)	0.655	
LVEF	<50%	37	445	1.25 (0.88–1.80)	0.216	0.005
	≥50%	16	1064	4.44 (2.25–8.77)	<0.001	
LDL-C	≥2.6	23	589	1.49 (0.86–2.59)	0.158	0.494
	<2.6	30	920	1.75 (1.17–2.60)	0.006	

Models adjust for age, sex, smoking, hypertension, diabetes, dyslipidemia, prior MI, prior stroke, target CTO artery, multi-vessel disease, statin, dual antiplatelet therapy, LVEF, creatinine, TG, HDL-C, CRP, FBG, and HbA1c. HR, hazard ratio; CI, confidence interval; Lp(a), Lipoprotein(a); CTO, chronic total occlusion; LVEF, left ventricular ejection fraction; PCI, percutaneous coronary intervention; MI, myocardial infarction; LDL-C, low-density lipoprotein cholesterol.

## Data Availability

The datasets used during the current study are available from the corresponding authors on reasonable request.

## Supplementary Materials

The following supporting information can be downloaded at: https://www.mdpi.com/article/10.3390/jcdd13070320/s1. Table S1: Subgroup analysis of association of Lp(a) and MACEs in participants with successful CTO PCI. Table S2: Associations between Lp(a) and clinical outcomes in participants with successful CTO PCI after excluded participants with follow-up ≤60 day (N = 1476). Table S3: Associations between Lp(a) and clinical outcomes in participants with successful CTO PCI after excluded participants with prior CABG (N = 1474). Table S4: Associations between Lp(a) and clinical outcomes in participants with successful CTO PCI after excluded participants with prior stroke (N = 1436). Table S5: Associations between Lp(a) and clinical outcomes in participants with successful CTO PCI additionally adjust for ApoA1 and ApoB100 (N = 1509).
