# Supplementary material for: Lipoprotein(a) and Adverse Outcomes After Successful Percutaneous Coronary Intervention for Chronic Total Occlusion: A Single-Center Retrospective Cohort Study
Source: J Cardiovasc Dev Dis. 2026 Jul 9;13(7):320. doi: 10.3390/jcdd13070320 (PMC13410022; doi:10.3390/jcdd13070320)
Supplement: Supplementary file 1 [file jcdd-13-00320-s001.zip › jcdd-4338854-supplementary.pdf]

Table S1. Subgroup analysis of association of Lp(a) and MACEs in participants with successful CTO PCI.

**Subgroups**		**MACEs**	**N**	**HR (95% CI)**	**P value**	**P for interaction**
Sex	Male	55	1,382	1.59 (1.17-2.16)	0.003	0.216
	Female	7	127	0.56 (0.23-1.37)	0.203	
Age	<60 years	21	765	1.86 (1.11-3.10)	0.018	0.484
	≥60 years	41	744	1.42 (0.99-2.03)	0.058	
Current Smoking	Yes	23	588	1.44 (0.89-2.34)	0.137	0.893
	No	39	921	1.48 (1.01-2.17)	0.044	
Prior MI	Yes	19	234	1.42 (0.67-2.97)	0.358	0.671
	No	43	1,275	1.50 (1.08-2.10)	0.016	
Diabetes	Yes	33	601	1.51 (1.00-2.28)	0.051	0.860
	No	29	908	1.52 (1.00-2.32)	0.051	
Hypertension	Yes	47	899	1.81 (1.29-2.55)	<0.001	0.033
	No	15	610	0.79 (0.38-1.66)	0.533	
LVEF	<50%	37	445	1.24 (0.87-1.77)	0.233	0.033
	≥50%	25	1,064	2.10 (1.25-3.54)	0.005	
LDL-C	≥2.6	25	589	1.32 (0.78-2.22)	0.305	0.353
	<2.6	37	920	1.66 (1.15-2.39)	0.007	

Models adjust for age, sex, smoking, hypertension, diabetes, dyslipidemia, prior MI, prior stroke, target CTO artery, multi-vessel disease, statin, dual antiplatelet therapy, LVEF, creatinine, TG, HDL-C, CRP, FBG, and HbA1c.

HR, hazard ratio; CI, confidence interval; Lp(a), Lipoprotein(a); CTO, chronic total occlusion; LVEF, left ventricular ejection fraction; PCI, percutaneous coronary intervention; MI, myocardial infarction; LDL-C, low-density lipoprotein cholesterol.

Table S2. Associations between Lp(a) and clinical outcomes in participants with successful CTO PCI after excluded participants with follow-up≤60 day (N = 1476).

	**Cardiovascular mortality**		**MACEs**	
	HR (95%CI)	P-value	HR (95%CI)	P-value
Per-SD increase in Log(Lp(a))	1.69 (1.16-2.46)	0.006	1.56 (1.12-2.17)	0.009
T1	Reference		Reference	
T2	1.18 (0.44-3.18)	0.748	1.55 (0.63-3.77)	0.337
T3	2.55 (1.03-6.28)	0.042	2.53 (1.09-5.88)	0.031
Lp(a)				
＜30 mg/dL	Reference		Reference	
30-50 mg/dL	1.36 (0.56-3.34)	0.499	1.06 (0.45-2.48)	0.890
≥50 mg/dL	2.49 (1.16-5.37)	0.020	2.20 (1.11-4.35)	0.024
Lp(a)				
＜50 mg/dL	Reference		Reference	
≥50 mg/dL	2.24 (1.12-4.50)	0.023	2.16 (1.16-4.02)	0.016

Models adjust for age, sex, smoking, hypertension, diabetes, dyslipidemia, prior MI, prior stroke, target CTO artery, multi-vessel disease, statin, dual antiplatelet therapy, LVEF, creatinine, TG, HDL-C, CRP, FBG, and HbA1c.

Table S3. Associations between Lp(a) and clinical outcomes in participants with successful CTO PCI after excluded participants with prior CABG (N = 1474).

	**Cardiovascular mortality**		**MACEs**	
	HR (95%CI)	P-value	HR (95%CI)	P-value
Per-SD increase in Log(Lp(a))	1.58 (1.16-2.16)	0.004	1.51 (1.13-2.00)	0.005
T1	Reference		Reference	
T2	1.33 (0.59-3.00)	0.491	1.59 (0.75-3.37)	0.231
T3	1.67 (1.24-5.72)	0.011	2.64 (1.28-5.44)	0.009
Lp(a)				
＜30 mg/dL	Reference		Reference	
30-50 mg/dL	1.39 (0.67-2.88)	0.383	1.18 (0.58-2.40)	0.642
≥50 mg/dL	2.31 (1.20-4.46)	0.012	2.16 (1.19-3.94)	0.012
Lp(a)				
＜50 mg/dL	Reference	0.512	Reference	
≥50 mg/dL	2.09 (1.14-3.85)	0.018	2.06 (1.18-3.60)	0.011

Models adjust for age, sex, smoking, hypertension, diabetes, dyslipidemia, prior MI, prior stroke, target CTO artery, multi-vessel disease, statin, dual antiplatelet therapy, LVEF, creatinine, TG, HDL-C, CRP, FBG, and HbA1c.

Table S4. Associations between Lp(a) and clinical outcomes in participants with successful CTO PCI after excluded participants with prior stroke (N = 1436).

	Cardiovascular mortality		MACEs	
	**HR (95%CI)**	***P*-value**	**HR (95%CI)**	***P*-value**
Per-SD increase in Log(Lp(a))	1.68 (1.19-2.35)	0.003	1.53 (1.13-2.07)	0.006
T1	Reference		Reference	
T2	1.18 (0.47-2.96)	0.726	1.48 (0.64-3.43)	0.356
T3	2.90 (1.26-6.67)	0.013	2.72 (1.24-5.94)	0.012
Lp(a)				
＜30 mg/dL	Reference		Reference	
30-50 mg/dL	1.46 (0.64-3.33)	0.364	1.12 (0.51-2.47)	0.771
≥50 mg/dL	2.64 (1.32-5.27)	0.006	2.26 (1.21-4.20)	0.010
Lp(a)				
＜50 mg/dL	Reference		Reference	
≥50 mg/dL	2.34 (1.25-4.38)	0.008	2.18 (1.23-3.86)	0.007

Models adjust for age, sex, smoking, hypertension, diabetes, dyslipidemia, prior MI, target CTO artery, multi-vessel disease, statin, dual antiplatelet therapy, LVEF, creatinine, TG, HDL-C, CRP, FBG, and HbA1c.

Table S5. Associations between Lp(a) and clinical outcomes in participants with successful CTO PCI additionally adjust for ApoA1 and ApoB100 (N = 1509).

	Cardiovascular mortality		MACEs	
	**HR (95%CI)**	***P*-value**	**HR (95%CI)**	***P*-value**
Per-SD increase in Log(Lp(a))	1.55 (1.12-2.13)	0.008	1.47 (1.09-1.97)	0.011
T1	Reference		Reference	
T2	1.26 (0.55-2.88)	0.581	1.57 (0.73-3.37)	0.245
T3	2.43 (1.10-5.32)	0.027	2.45 (1.16-5.16)	0.018
Lp(a)				
＜30 mg/dL	Reference		Reference	
30-50 mg/dL	1.30 (0.62-2.72)	0.491	1.11 (0.54-2.28)	0.768
≥50 mg/dL	2.13 (1.09-4.16)	0.028	1.97 (1.07-3.64)	0.030
Lp(a)				
＜50 mg/dL	Reference		Reference	
≥50 mg/dL	1.96 (1.05-3.64)	0.035	1.91 (1.08-3.36)	0.026

Models adjust for age, sex, smoking, hypertension, diabetes, dyslipidemia, prior MI, prior stroke, target CTO artery, multi-vessel disease, statin, dual antiplatelet therapy, LVEF, creatinine, TG, HDL-C, CRP, FBG, HbA1c, ApoA1, and ApoB100.
